# Heterogeneity of the horizontal environment drives community assemblages and species coexistence of prokaryotic communities in cold seep sediments

**DOI:** 10.3389/fmicb.2025.1687453

**Published:** 2025-11-20

**Authors:** Qixuan Wu, Jingchun Feng, Yongji Huang, Song Zhong, Cun Li, Si Zhang

**Affiliations:** 1 Guangdong Basic Research Center of Excellence for Ecological Security and Green Development, School of Ecology, Environment and Resources, Guangdong University of Technology, Guangzhou, China; 2 Southern Marine Science and Engineering Guangdong Laboratory (Guangzhou), Guangzhou, China; 3 University of Chinese Academy of Sciences, Beijing, China; 4 South China Sea Institute of Oceanology, Chinese Academy of Sciences, Guangzhou, China

**Keywords:** cold seep sediment, horizontal environmental heterogeneity, community diversity, community assembly, species coexistence

## Abstract

**Introduction:**

Microorganisms play important roles in methane oxidation and carbon turnover of cold seep ecosystem. Although the microbial diversity of cold seep sediments has been reported, the community assembly processes, species coexistence patterns, and their underlying drivers across horizontal gradients of distinct cold seep habitats remain largely unexplored. To address this gap, we conducted a comprehensive investigation of prokaryotic communities in various habitats of the Haima cold seeps, aiming to elucidate the mechanisms governing microbial community construction in this sedimentary environment.

**Methods:**

Sediment samples were collected from the Haima cold seep and subsequently analyzed through an integration of geochemical measurements and 16S rRNA gene sequencing.

**Results:**

The prokaryotic community at the methane seep site exhibited lower *α*-diversity than those at other sites. Halobacterota dominated the methane seep site, whereas higher abundances of Chloroflexi and Asgardarchaeota were observed in the faunal sites. The assembly process of the bacterial community in the methane seep site was primarily governed by stochastic processes, while the archaeal community was mainly formed by deterministic processes. In faunal sites, both stochastic and deterministic processes influenced prokaryotic community assembly. Heterogeneity of the horizontal environment such as the content of CH_4_, Ba^2+^, total inorganic carbon, and SO_4_^2−^ significantly influenced prokaryotic community diversity and governed the community assembly. Additionally, co-occurrence network analysis revealed higher connectivity and more complex species interactions in the bacterial network at the methane seep site compared to other sites; the opposite trend was observed for archaea.

**Discussion:**

This study demonstrated that environmental heterogeneity is a key determinant of prokaryotic community diversity and composition in the cold seep, driving distinct community assembly and species coexistence patterns across different habitats.

## Graphical abstract

**Figure:**
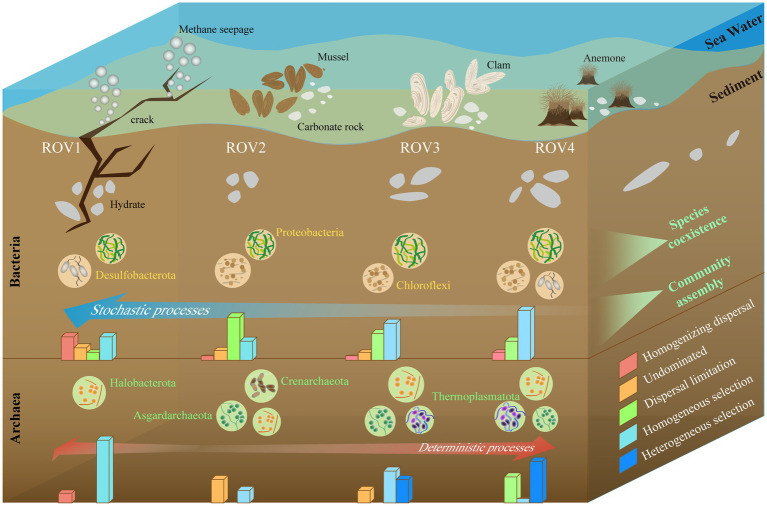

## Highlights

- Habitat heterogeneity affected the species coexistence and community assembly.
- CH_4_ was the key driving factor of prokaryotic community diversity and assembly.
- The assembly of prokaryotic communities were mainly stochastic processes.
- Bacteria and archaea showed different coexistence patterns in seepage area.

## Introduction

Cold seeps are deep-sea phenomena caused by fluids rich in methane, hydrogen sulfide, and other hydrocarbons released from seabed sediments (Lee et al., 2014; Feng et al., 2018). In this type of seepage environment, sulfate-driven anaerobic oxidation of methane is the predominant process mediated by methanotrophic archaea and sulfate-reducing bacteria (SRB) (Knittel and Boetius, 2009). With the consumption of methane and sulfate, this biochemical process releases abundant dissolved bicarbonate and hydrogen sulfide (Lin et al., 2022; Gong et al., 2023). These processes provide carbon and energy sources for the surrounding prokaryotic and macrofaunal communities, derived from the thriving cold seep chemosynthetic ecosystem (Lyu et al., 2023).

During ecological succession at a cold seep, crevices appear in the sediments as a result of methane seepage; this continuous seepage of methane attracts microorganisms capable of participating in methane metabolism to colonize the site. Abundant benthic faunas such as deep-sea white clams and sea anemones appear at seep sites after initial microbial colonization, and mussels gradually dominate there when methane seepage weakens. Carbonate rocks develop at older, late-stage seeps (Feng et al., 2023a). The intensity of methane seepage varies in successional periods, resulting in the formation of different habitats. In Haima cold seeps located in the South China Sea as described below, remotely operated vehicle observations have confirmed the presence of typical cold seep biota including mussels, tubeworms, clams, and microbial mats (Hu et al., 2019; Chen et al., 2023). Different methane seepage rates and densities of various organisms were also observed in different habitats (Xu et al., 2020). Correspondingly, dense populations of mussels, clams, shrimps, and deep-sea crabs appeared in strongly bubbling areas, while large amounts of carbonate rocks and dead shells existed in the weak seepage zone (Liang et al., 2017; Xu et al., 2020; Feng et al., 2023b). Therefore, the different stages of cold seep succession harbor a variety of habitats (Chen et al., 2023; Niu et al., 2023).

Niche and neutral theories have received considerable of attention in ecology (Whitfield, 2002; Wennekes et al., 2012). Niche theory advocates that deterministic processes such as environmental filtering (e.g., temperature, pH, moisture, and salt), interspecies interactions (e.g., competition, predation, mutualisms, and trade-offs) determine community composition, abundance, and distribution. Homogeneous and heterogeneous environmental pressures lead to more similar (Homogeneous selection) and dissimilar (Heterogeneous selection) structure among communities, respectively. The neutral theory argues that species are functionally equal and highly affected by stochastic processes such as colonization, extinction, and speciation (Chase and Myers, 2011; Zhou and Ning, 2017; Ning et al., 2020). These ecological processes are summarized as selection, dispersal, speciation or diversification, and ecological drift, which individually or collectively drive the community assembly of a local microbial community. Even within a system, the relative impacts of these ecological processes in different communities vary greatly (Stegen et al., 2015). Species coexistence occurs when interspecific competition is weaker than intraspecific competition, and promotes niche differentiation (Gravel et al., 2011). Ecological co-occurrence networks represent microbial species as nodes and interspecific interactions as edges. These networks can be used to infer potential species coexistence and interactions, such as competition, predation, mutualism, and commensalism, within a community. Methane and related geochemical variables fundamentally shape both faunal assemblages and key microbial communities. The progression from strong methane seepage to distinct stages of faunal colonization reflects the environmental heterogeneity of cold seep ecosystems (Chen et al., 2023). Since environmental conditions at cold seeps vary successionally, the associated microbial community diversity, assembly, and species coexistence patterns may differ across stages.

Haima cold seep is one of the two active seep sites in the South China Sea at present, and the other one is the F Site cold seep (Feng et al., 2018). At present, Haima cold seep has been regarded as a research hotspot. It was revealed that the ecological and geochemical gradients differed in distinct areas of the Haima cold seeps, and the distribution of benthic faunas was associated with methane and sulfides (Xu et al., 2020). Although many studies about microorganisms have been conducted on Haima cold seeps, these studies have mainly focused on the distribution and diversity of the microbial communities. It has been reported that there is a preference distribution of specific ANME at different depths in Haima cold seeps sediments (Niu et al., 2017). A study also suggested an interaction between environmental conditions and microorganisms that might have played an important role in the carbon and sulfur cycles (Chen et al., 2023). Moreover, several studies on Haima cold seeps have shown that methane fluids promote microbial aggregation and evolution in sediments (Niu et al., 2017; Dong et al., 2023; Zhong et al., 2023). Environmental heterogeneity in different areas of Haima cold seeps system affected the composition of microbial communities, which even was associated to mineral processes there (Liang et al., 2023). However, how horizontal environmental heterogeneity affects community assembly and species coexistence in different areas of Haima cold seeps was still unclear.

In this study, an investigation of the geochemical properties and microbial community diversity of sediments from four habitats in Haima cold seeps in the South China Sea was carried out. Specifically, 16S ribosomal RNA (rRNA) gene amplification sequencing was applied to measure the bacterial and archaeal communities. Null models and co-occurrence networks were used to characterize the assembly processes and species coexistence patterns of prokaryotic communities. The purposes of this study are to: (i) explore the horizontal distribution of the prokaryotic communities in different cold seep habitats, (ii) clarify community assembly processes and species coexistence patterns along the horizontal scale of cold seep sediments, and (iii) analyze the important environmental factors that drive community diversity, assembly processes and species coexistence.

## Materials and methods

### Collection of sediment samples and geochemical analysis

The sediments were obtained using a pushcore controlled by a remotely operated vehicle at four sites of Haima cold seeps (16°43′N, 110°28′E) located in the South China Sea during May 2023. Sediment samples were collected from depths of 0–5, 5–10, 10–15, 15–20, 20–25, 25–30, and 30–35 cm and were stored at −80 °C until analysis. The porewater was extracted from sediments by Rhizon samplers and kept at 4 °C until analysis. The four sampling sites were ROV1, ROV2, ROV3 and ROV4, respectively, (Supplementary Figure S1). Of the four sites, the ROV1 site was identified as a methane seep area with strong methane seepage. The ROV2, ROV3, and ROV4 sites were defined as faunal areas with weak methane seepage and were, respectively, colonized by abundant mussels, clams, and sea anemones. The distance between the four sites was 4.6–12.2 km.

The geochemical parameters measured included total inorganic carbon (TIC), total organic carbon (TOC), and concentrations of CH_4_, SO_4_^2−^, Cl^−^, Ba^2+^, K^+^, Ca^2+^, Mg^2+^, Fe^3+^, Cu^2+^, and Mn^2+^. The Headspace Equilibrium method was used to measure CH_4_ concentrations in the sediments. Specifically, 5 g of sediment was transferred into a glass vial, and 5 mL of NaOH (5% w/w) was immediately added. Then the glass vial was sealed with butyl rubber stopper and aluminum rolled sheet and shaken for 10 min to achieve methane equilibrium between the aqueous and gas phases. The CH_4_ concentrations of the samples were measured by gas chromatography (Trace 1,300, Thermo Fisher, Waltham, MA, United States). Concentrations of TOC, TIC and SO_4_^2−^, Cl^−^, Ba^2+^, K^+^, Ca^2+^, Mg^2+^, Fe^3+^, Cu^2+^, and Mn^2+^ in sediments were measured using the porewater extracted from the sediments. TIC and TOC were measured by a TOC analyzer (Shimadzu TOC-L, Kyoto, Japan). The concentrations of SO_4_^2−^ and Cl^−^ were detected by ion chromatography (Thermo Fisher AQ-1200). An inductively coupled plasma-optical emission spectrometer (ICP-OES, Thermo Fisher iCAP 7,000 series) was used to determine the concentrations of Ca^2+^, Mg^2+^, Ba^2+^, K^+^, Fe^3+^, Cu^2+^, and Mn^2+^. All testing procedures followed manufacturer’s instructions.

### DNA extraction, PCR amplification, 16 s rRNA sequencing

Total genomic DNA was extracted from sediments using a Magnetic Soil and Stool DNA Kit (Tiangen, DP712-02, Tianjin, China). The quality and concentration of the extracted DNA were assessed using qubit fluorometric quantification (Thermo Scientific, Thermo Fisher Scientific Corp.). Forward primer 341F (5’-CCT ACG GGN GGC WGC AG − 3′) and reverse primer 806R (5′- GGA CTA CHV GGG TWT CTA AT −3′) (Hugerth et al., 2014) were used to amplify the V3–V4 region of the bacterial 16S rRNA gene. Archaeal 16S rRNA gene was amplified by forward primer Arc349F (5′-GYG CAS CAG KCG MGA AW-3′) and reverse primer Arc806R (5′-GGA CTA CNS GGG TMT CTA AT-3′) (Takai and Horikoshi, 2000). Six specific bp barcodes were tagged on the primers to distinguish the sequences of each sample in the mixed pools used for Illumina sequencing. Next, PCR amplification was performed using the following settings: pre-denaturation at 95 °C for 3 min, 30 cycles consisting of denaturation at 95 °C for 15 s, annealing at 53 °C for 15 s, and extension at 72 °C for 30 s, and a final extension at 72 °C for 10 min. The amplified PCR products were purified with DNA Clean Beads (Vazyme, N411-01, China). An equal quantity of DNA from each sample was mixed for amplicon library construction. Amplicon libraries were sequenced on a Novasep-PE250 platform (Illumina Inc., San Diego, CA, United States). All raw sequences in this study have been deposited at the NCBI Sequence Read Archive under the accession number PRJNA1230284.

### Data processing and bioinformatics analysis

The bioinformatics tool Quantitative Insights into Microbial Ecology version 2 (2022.8) was used for data processing. The raw data were split into the amplicon sequences of individual samples by the plugin cutadapt based on each unique barcode. The plugin dada2 was used to complete the quality control of sequences. The analysis utilized 40 threads. First, 20 bp from both the forward and reverse primer were removed to eliminate non-biological regions. The low-quality ends of the reads were trimmed, retaining 240 bp high-quality reads. Next, the high-quality reads with 100% similarity were clustered to form amplicon sequence variants (ASVs), and the feature tables and representative sequences were obtained. To further clarify the representative sequences, the feature-classifier plugin and the Silva reference database (version 138.1) were used to perform taxonomic annotations and produce a taxonomy table. Finally, the phylogenetic trees were constructed using the Phylogeny plugin. In detail, multiple sequence alignment was performed using MAFFT, followed by the application of the default masking strategy to remove low-quality alignment regions. Finally, phylogenetic trees were constructed with FastTree.

### Statistical analysis

The following data analysis was performed by R software (vers. 4.3.0). Before the analysis, the taxonomy table was rarefied with the rrarefy function (“vegan” package) (vers. 2.6–4). The *α*-diversities of the bacterial and archaeal communities were calculated using the “vegan” package in R (Dixon, 2003), including abundance-based coverage estimator ACE, Observed ASVs, Phylogenetic diversity, Shannon diversity and Good’s coverage. Furthermore, the least significant difference method was used (“agricolae” package) to calculate the significant differences between samples (Meier, 2006). Principal coordinate analysis (PCoA) based on Bray-Curtis distance was performed using the “vegan” package to show the differences in the prokaryotic community between sites. The relative abundance of community compositions at the phylum and genus level was represented by a stack histogram and heatmap, respectively. Linear discriminant analysis effect size was performed among sites to obtain representative microorganisms with statistical differences and the extent of their effects (Chang et al., 2022). The unique and shared ASVs between sites were calculated and were visualized using a petal chart.

Based on the evolutionary distance of the phylogenetic tree, a null model was applied to calculate the mean-nearest-taxon distance, which was transformed into the nearest-taxon index (NTI) to reflect the closest interspecific affinities. To obtain the *β*-nearest-taxon index (βNTI) and describe the spatial and temporal succession of community phylogenetic composition, the β-mean-nearest-taxon distance (βMNTD) was calculated using the comdistnt function in R. The results were divided according to the following conditions: when |βNTI| > 2, it indicates that the community assembly is controlled by a deterministic process. When βNTI>2, this indicates heterogeneous selection, while βNTI<−2 indicates that the turnover in community composition is lower than expected by chance, which is interpreted as homogeneous selection. When |βNTI| < 2, it indicates that a stochastic process is occurring. In this premise, when RCbray>0.95, this means that the Raup-Crick (RC) distance between species is relatively large, which is defined as the dispersal limitation. When RCbray<−0.95, this indicates that the RC distance between species is relatively small and tends to be homogeneous, which indicates homogenizing dispersal is occurring. Additionally, when |βNTI| < 2 and |RCbray| < 0.95 are met at the same time, indicating low turnover and a small RC distance, the process is considered to be undominated (e.g., weak selection, weak dispersal, diversification, and drift are occurring). Based on the above approach, we calculated the proportion of the same ecological processes across different depths at each site, representing the distribution of ecological factors influencing the community assembly processes (Stegen et al., 2013). Spearman’s correlation coefficient was calculated and a Mantel test (“linkET” package) was conducted to analyze the correlation between the composition of the microbial community and environment parameters (Legendre and Fortin, 2010). Random Forest prediction was carried out in the “rfPermute” package (Han and Trimi, 2024) to evaluate the influence of environmental factors such as concentrations of CH_4_, SO_4_^2−^, Cl^−^, Ca^2+^, Mg^2+^, Ba^2+^, K^+^, Fe^3+^, Cu^2+^, and Mn^2+^ on microbial distribution at the four stations (ROV1–ROV4).

The bacterial and archaeal communities at each site were used to construct the microbial co-occurrence networks for each site. The ASVs with relative abundance >0.005 were extracted using the “psych” and “igraph” packages. The correlations between individual ASVs were calculated using Spearman’s correlation coefficients and the “Benjamini–Hochberg” method was applied to correct the *p* values of the correlation coefficients. The obtained networks were visualized in Gephi software, and the basic topological properties of the networks were derived, including average degree, average clustering coefficient, average path length and modularity. Average degree indicates the average number of connections between nodes. The clustering coefficient of a node refers to the ratio of the number of connected edges between its neighbors to the maximum number of possible connected edges between these neighbors. Average clustering coefficient is the average of the clustering coefficients of all nodes in the network. Average path length represents the average of the distance between any two nodes. Modularity represents the number and structure of modules composed of nodes in a network.

The visualization of the above analysis was completed in R and Origin, Version 2024 (OriginLab Corporation, Northampton, MA, USA). The environmental factors, *α*-diversity boxplots, heatmaps, phylum community composition and community assembly stacking bar charts, representative microorganism bar charts, NTI, βNTI scatter plots, and network topology bar charts were all drawn using Origin software. The results of PCoA, a Mantel test, and Random Forest were all visualized using the “ggplot2” package of R software, and a petal diagram was drawn using the “plotrix” package.

## Results

### Horizontal geochemical heterogeneity of sediments

**Figure 1:**
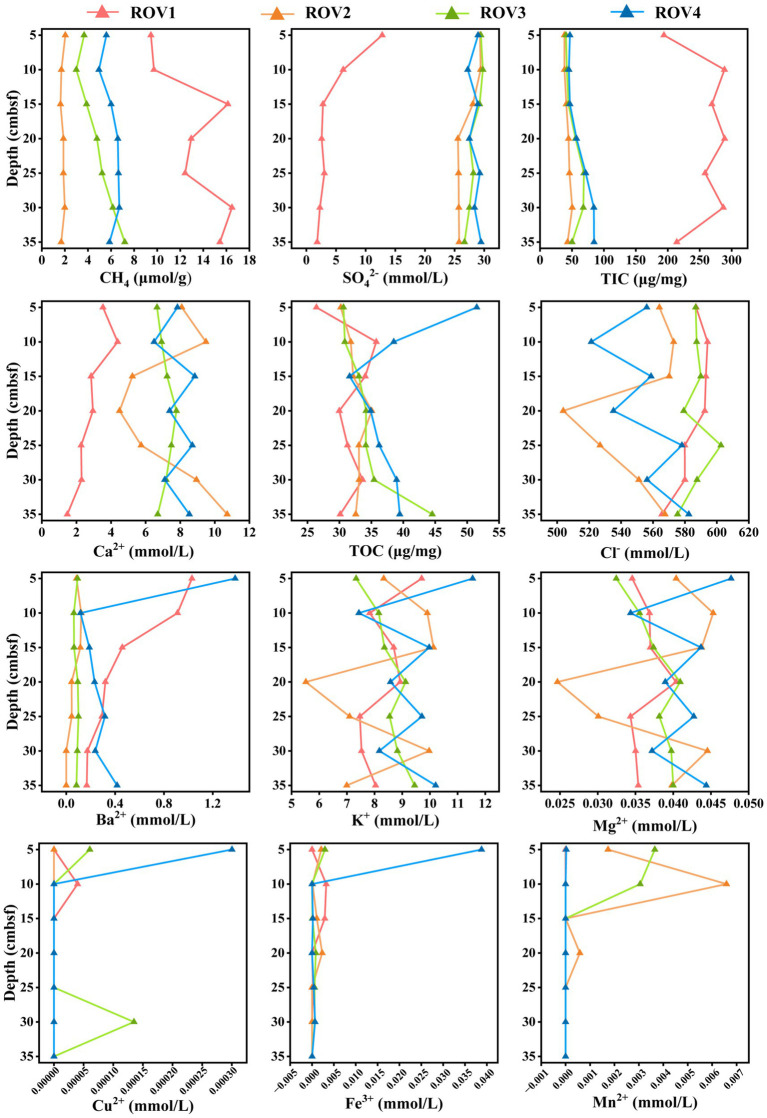
Geochemical properties of sediment samples from four sites of Haima cold seeps in the South China Sea. Specifically, concentrations of CH_4_ of sediments and concentrations of TIC, TOC, and SO_4_^2−^, Cl^−^, Ba^2+^, K^+^, Ca^2+^, Mg^2+^, Fe^3+^, Cu^2+^, and Mn^2+^ of porewater from sediments were measured.

The environmental factors of sediments at different Haima cold seeps are shown in Figure 1 and Supplementary Table S1. The geochemical properties changed along the horizontal profiles of sediments. Sediments from site ROV1 exhibited higher concentrations of CH_4_, TIC, and Ba^2+^ but lower concentrations of TOC, SO_4_^2−^, and Ca^2+^ compared to the other three sites. The concentrations of K^+^, Mg^2+^, Fe^3+^, Cu^2+^, and Mn^2+^ were not significantly different between sites, and the concentrations of Fe^3+^, Cu^2+^, and Mn^2+^ were extremely low.

Significant differences in geochemical properties were found between the methane seep area (ROV1) and the areas with different macrofaunal assemblages (ROV2, ROV3, and ROV4), which were attributed to the different biogeochemical processes. The abundance of methane drives the microbe-mediated anaerobic oxidation of methane (AOM) in ROV1 sediments and coupled sulfate reduction to generate large amounts of HCO_3_^−^. As a result, relatively higher CH_4_ and TIC concentrations and a lower sulfate concentration were observed in ROV1 sediments. The bicarbonate produced by the AOM increased the alkalinity of the surrounding porewater and dissolved bicarbonate combined with Ca^2+^ to form authigenic carbonates (Akam et al., 2023; Gong et al., 2023). In short, the geochemical discrepancies were shown according to biogeochemical conditions at different locations of Haima cold seeps.

### Differences in prokaryotic community diversity of horizontal sediments

**Figure 2:**
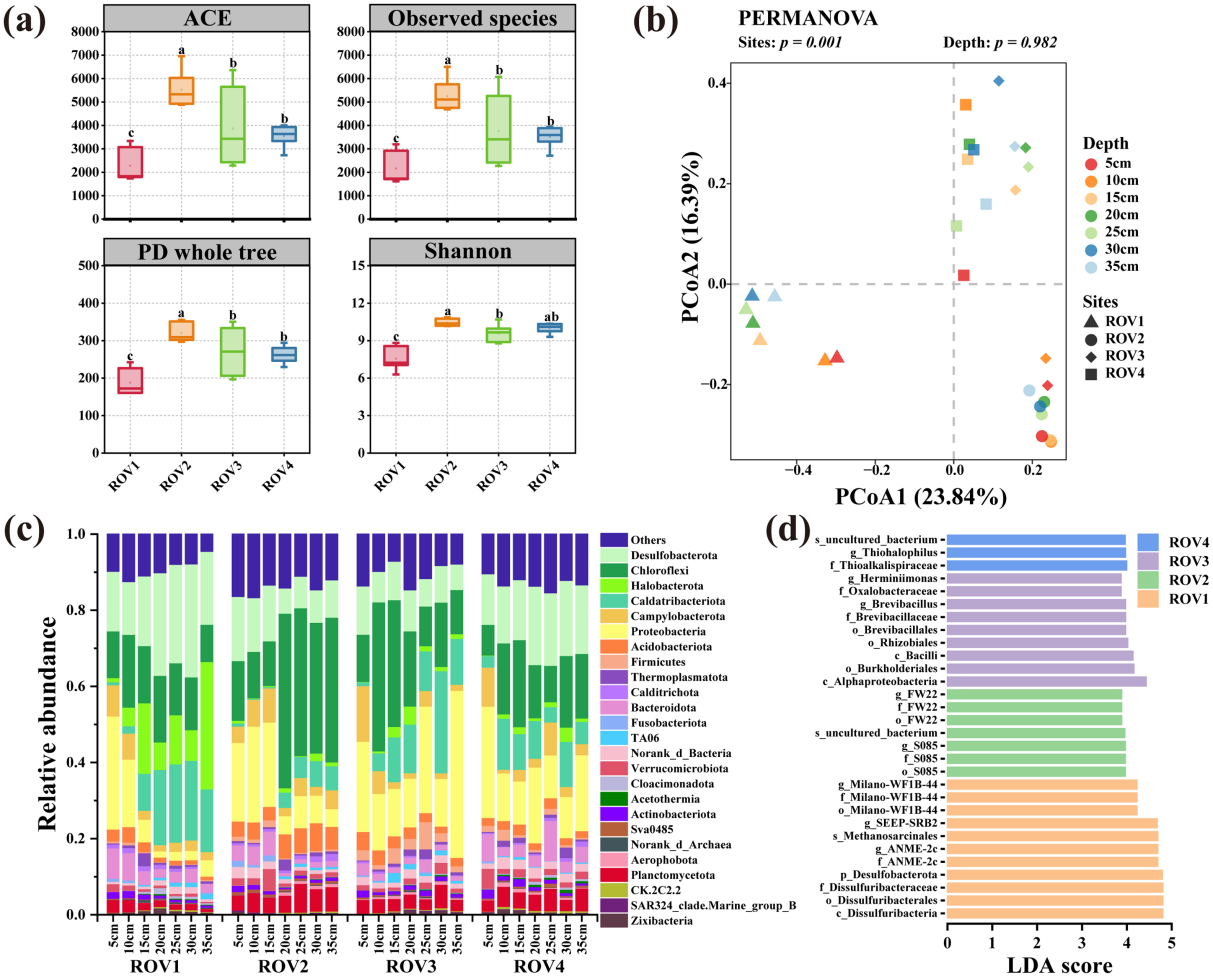
Horizontal community composition of bacteria for the sites ROV1, ROV2, ROV3, and ROV4: **(a)** horizontal *α*-diversity; letter inconsistencies between the data indicate significant differences between the sites (*p* < 0.05; least significant difference test); **(b)** principal coordinate analysis showing differences in community composition between sites; **(c)** relative abundance of microbial communities at the phylum level at sites; **(d)** linear discriminant analysis effect size showing representative microorganism microorganisms in sediment at different sites. LDA, linear discriminant analysis; PCo1 and PCo2, principal coordinates 1 and 2, respectively.

**Figure 3:**
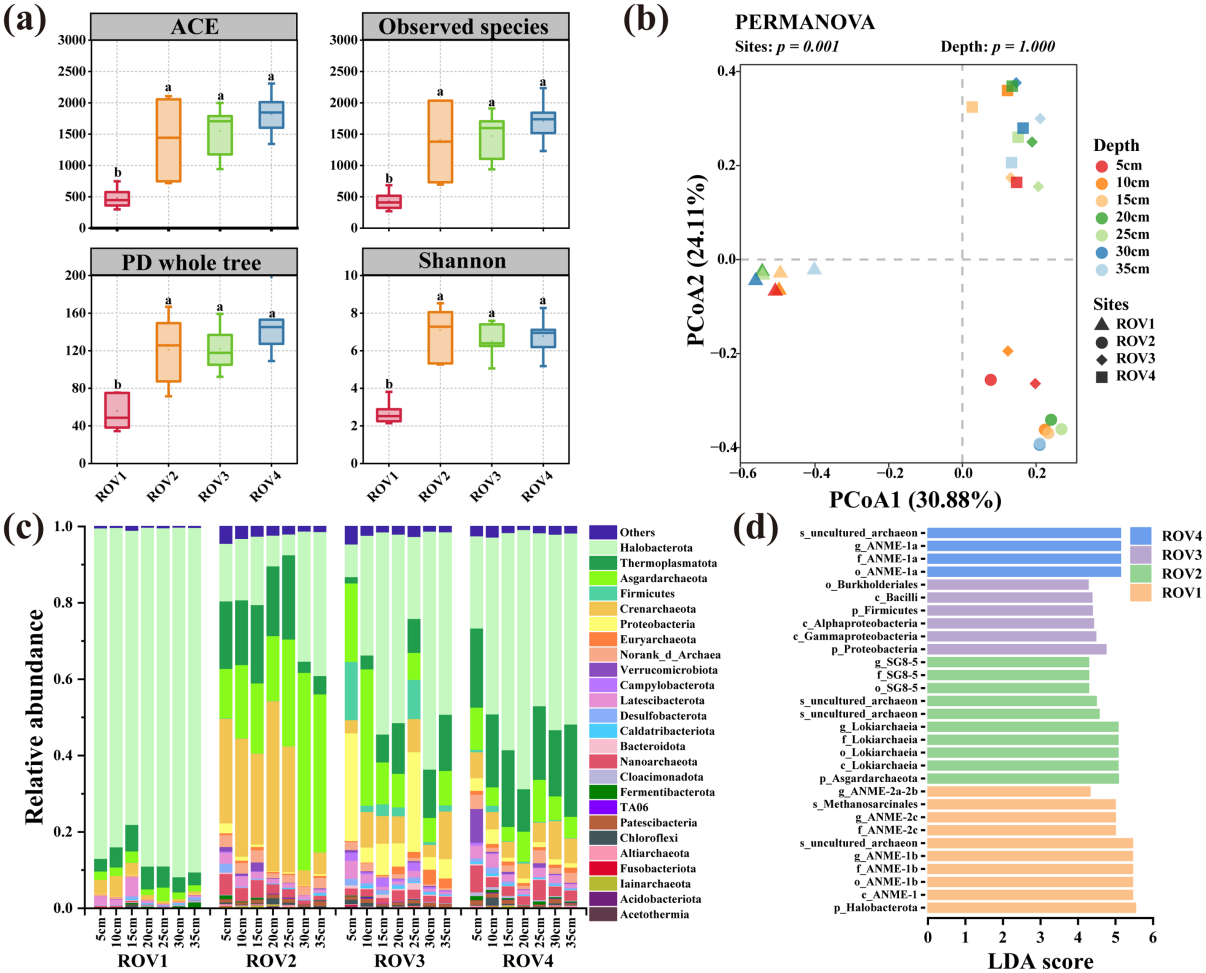
Horizontal community composition of Archaea for the sites ROV1, ROV2, ROV3, and ROV4: **(a)** horizontal α-diversity; letter inconsistencies between the data indicate significant differences between the sites (*p* < 0.05; least significant difference test); **(b)** principal coordinate analysis showing differences in community composition between sites; **(c)** relative abundance of microbial communities at the phylum level at sites; **(d)** linear discriminant analysis effect size showing representative microorganisms in sediment at different sites. LDA, linear discriminant analysis; PCo1 and PCo2, principal coordinates 1 and 2, respectively.

A total of 4,016,684 bacterial and 3,288,215 archaeal high-quality sequences were obtained after merging and filtering the raw data of 16S rRNA genes of bacteria and archaea from 28 sediment samples. The bacterial and archaeal reads were clustered to generate 54,081 and 20,180 ASVs, respectively. The average value of Good’s coverage (Supplementary Figures S2a,b) in each site was greater than 0.99 and the rarefaction curves (Supplementary Figures S2c,d) reached a plateau, indicating that the sequencing depth of all samples was sufficient to capture the majority of microbial diversity. The *α*-diversities of bacterial and archaeal communities across the sampling sites are shown in Figures 2a, 3a, and Supplementary Table S2. The ACE, Observed ASVs, Phylogenetic diversity, and Shannon indices exhibited distinct profiles of microbial α-diversity in different sediments. Bacterial α-diversity was highest at the ROV2 site and lowest at ROV1, while archaeal α-diversity was also lowest at ROV1 and differed significantly from the other three sites.

The PCoA analysis showed that clustering patterns for the ROV1-4 bacterial and archaeal communities were concentrated in the third, fourth, first, and first quadrants, respectively. Permutational multivariate analysis of variance revealed significant differences among communities at different sites (*p* < 0.05), with those from the same site clustering together and those from different sites being well-separated. The first two principal coordinates explained 40.23 and 54.99% of the variance in the bacterial and archaeal communities, respectively (Figures 2b, 3b). A petal chart based on ASV composition revealed that samples grouped by site displayed more shared ASVs than those grouped by depth in both bacterial and archaeal communities (Supplementary Figure S3). The compositions of the bacterial (Figure 2c) and archaeal (Figure 3c) communities at each site were analyzed at the phylum level.

In the bacterial community, Desulfobacterota (13.8–29.7%) was more abundant at ROV1 than at other sites. Chloroflexi (11.8–45.9%) was the dominant phylum at ROV2. Meanwhile, ROV3 was dominated by relatively high abundances of Chloroflexi (10.4–39.2%) and Proteobacteria (12.5–43.9%), while Proteobacteria (10.9–29.3%), Chloroflexi (8.1–22.9%) and Desulfobacterota (13.3–20.6%) were dominant at ROV4. In the archaeal community, Halobacterota (77–91.5%) was the main group at ROV1. Asgardarchaeota (12.9–51.7%), Crenarchaeota (4.0–44.5%), and Halobacterota (5.5–37.7%) were the predominant phyla at ROV2. Halobacterota, Thermoplasmatota, and Asgardarchaeota were the dominant taxa of both ROV3 (ranges: 8.5–62.3, 1.7–14.7, and 6.4–35.7%, respectively) and ROV4 (ranges: 24.1–67.9,11.1–24.2, and 3.7–11.1%, respectively). However, ROV3 had a higher relative abundance of Firmicutes (0.7–15.2%) and Proteobacteria (3.3–28.2%) than ROV4. In addition, community composition at the genus level was also investigated (Supplementary Figure S4). In the bacterial community, *JS1* was dominant at ROV1, ROV3, and ROV4. Anaerolineaceae was dominant in ROV1, ROV2, and ROV3. In addition, SEEP-SRB1 specifically dominated in ROV1, while Dehalococcoidia was also relatively abundant at ROV2. In the archaeal community, ANME-1b (61.0%) and ANME-2c (18.6%) dominated at ROV1, while *Lokiarchaeia* and *Bathyarchaeia* were dominant at ROV2. In addition, ANME-1a (26.9 and 26.8%, respectively) was primarily found at ROV3 and ROV4. The linear discriminant analysis effect size (LEfSe) method was used to compare ASVs between sites and identify representative microorganisms in bacterial (Figure 2d) and archaeal (Figure 3d) communities. The results demonstrated distinct representative microorganisms across the four sampling sites. In the bacterial community, the class Dissulfuribacteria accounted for a higher proportion at ROV1, whereas the order S085 characterized the community at ROV2. Class Alphaproteobacteria was enriched at ROV3, and ROV4 was dominated by the family Thioalkalispiraceae. In the archaeal community, the phylum Halobacterota accounted for a higher proportion at ROV1, and the phylum Asgardarchaeota was significantly enriched at ROV2. The archaeal community at ROV3 was characterized by the phylum Proteobacteria, while ANME-1a was the dominant taxon at ROV4.

### The key environmental factors affect prokaryotic community diversity

**Figure 4:**
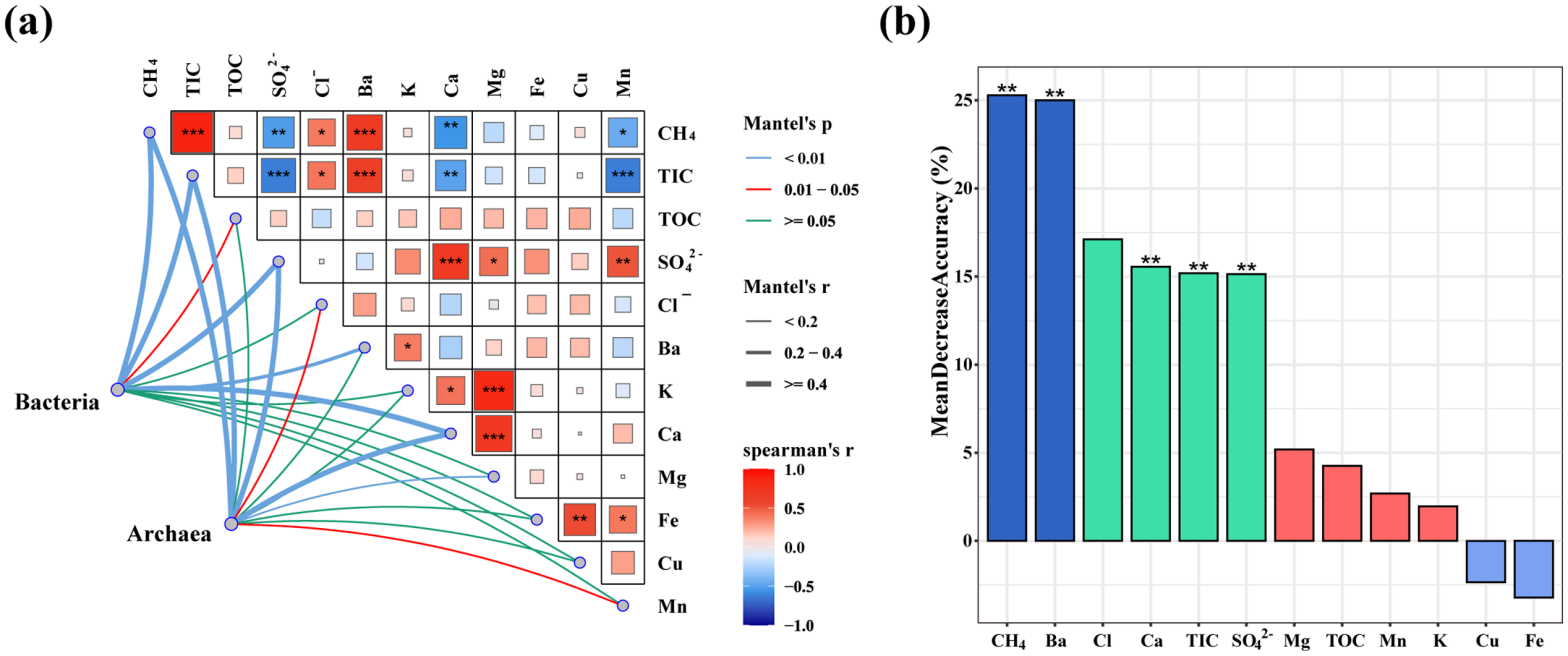
Horizontal environmental heterogeneity leads to prokaryotic community diversity: **(a)** Mantel test for analyzing the correlation between environmental parameters and community composition of prokaryotes; **(b)** Random Forest mean predictions of the impact of environmental variables on different sites. TIC, total inorganic carbon; TOC, total organic carbon.

To detect the influence of environmental variables on prokaryotic communities, a Mantel test (Figure 4a) and Random Forest model analysis (Figure 4b) were performed which depended on prokaryotic communities and environmental parameters. Mantel test results showed that environmental factors that had significant effects on the diversity of bacterial and archaeal communities were CH_4_, TIC, SO_4_^2−^, Ca^2+^, Ba^2+^, and Mg^2+^ (*p* < 0.01, r > =0.4). Random Forest results showed that the concentrations of CH_4_, Ba^2+^, Ca^2+^, SO_4_^2−^, and TIC had significant effects on the microbial community structure at different sites (p < 0.01); the Mean Decrease Accuracy value of CH_4_ was the highest (25.29%), indicating that methane had the most significant effect on the structure of the microbial community.

### Different community assembly processes of prokaryotic communities

**Figure 5:**
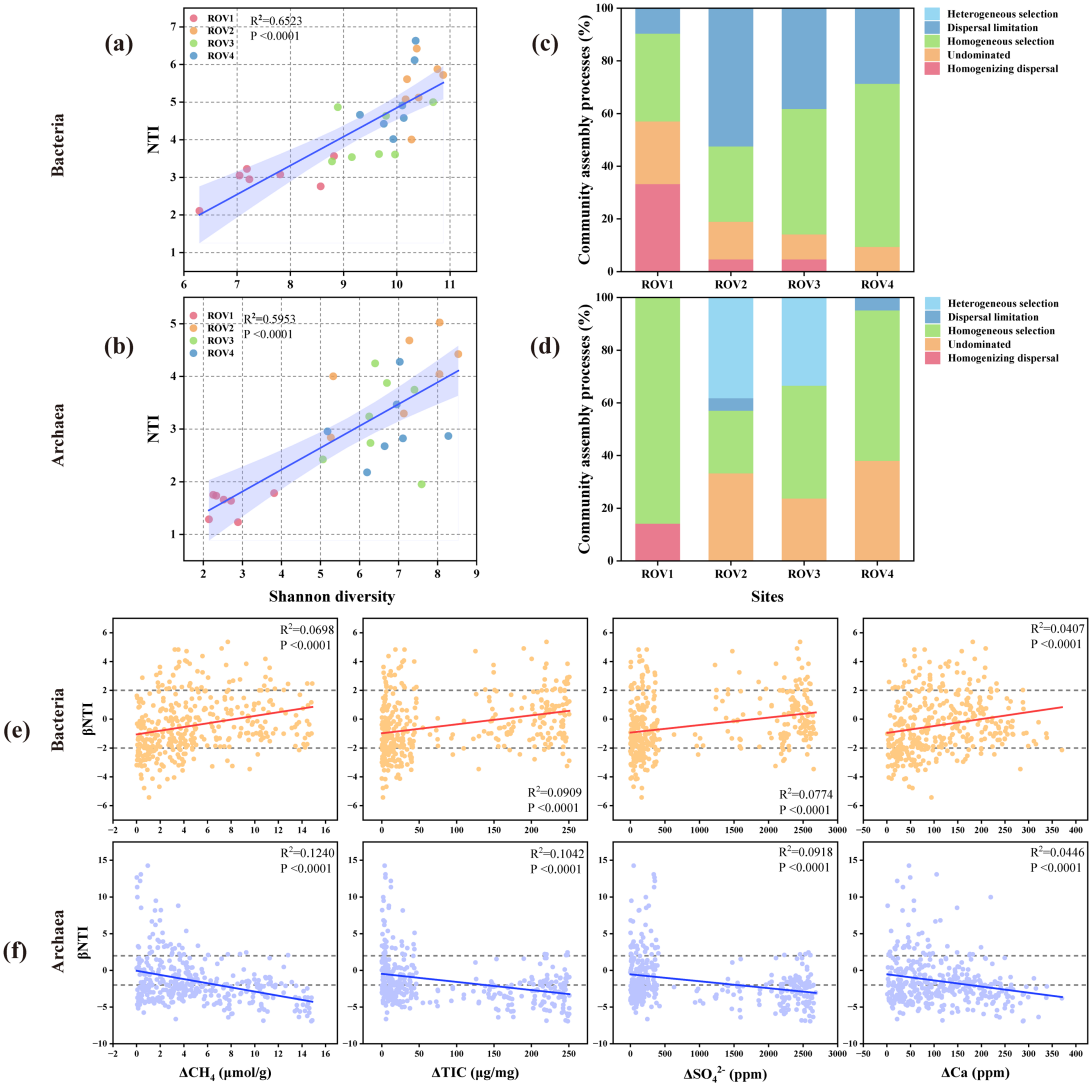
Community assembly mechanism of bacteria and archaea along the horizontal dimension for the sites ROV1, ROV2, ROV3, and ROV4: variation of **(a)** bacterial and **(b)** archaeal community nearest-taxon index (NTI) with the Shannon index; **(c)** bacterial and **(d)** archaeal community assembly mechanism at different sites; variation of *β*-NTI in **(e)** bacterial and **(f)** archaeal communities with environmental factors. TIC, total inorganic carbon.

To explore the assembly process of prokaryotic communities at different sites, the assembly mechanism of bacterial and archaeal communities was predicted across the different sites based on a null model (Figure 5). The NTI and Shannon diversity indices were positively correlated, suggesting that the community phylogenetic structure varied with community diversity (Figures 5a,b). Bacterial βNTI was positively correlated with differences in environmental parameters of CH_4_, TIC, SO_4_^2−^, and Ca^2+^ (*p* < 0.001; Figure 5e), while archaeal βNTI was negatively correlated with differences in environmental parameters (*p* < 0.001; Figure 5f). These parameters were important factors affecting community assembly. Stochastic processes accounted for a greater proportion in bacterial community assembly (Figure 5c), while both deterministic and stochastic processes contributed to the archaeal community assembly (Figure 5d).

In the assembly process of the bacterial community, the community of the ROV1 site was primarily influenced by homogenizing dispersal (33.3) along with undominated (23.8%) and homogeneous selection (33.3%). In contrast, dispersal limitation (28.6–52.4%) and homogeneous selection (28.6–61.9%) played more important roles at other sites. For archaeal communities, deterministic processes (homogeneous and heterogeneous selection processes) dominated the communities across all sites. Especially in communities of ROV1, homogeneous selection (85.7%) almost completely dominated the archaeal community. The archaeal communities at ROV2 and ROV3 were jointly affected by homogeneous selection (23.8–42.8%), homogeneous selection (33.3–38.1) and undominated (23.8–33.3%). In the community of ROV4, homogeneous selection (57.1%) and undominated (38.1%) were the main driving forces.

### Co-occurrence networks and topological properties

**Figure 6:**
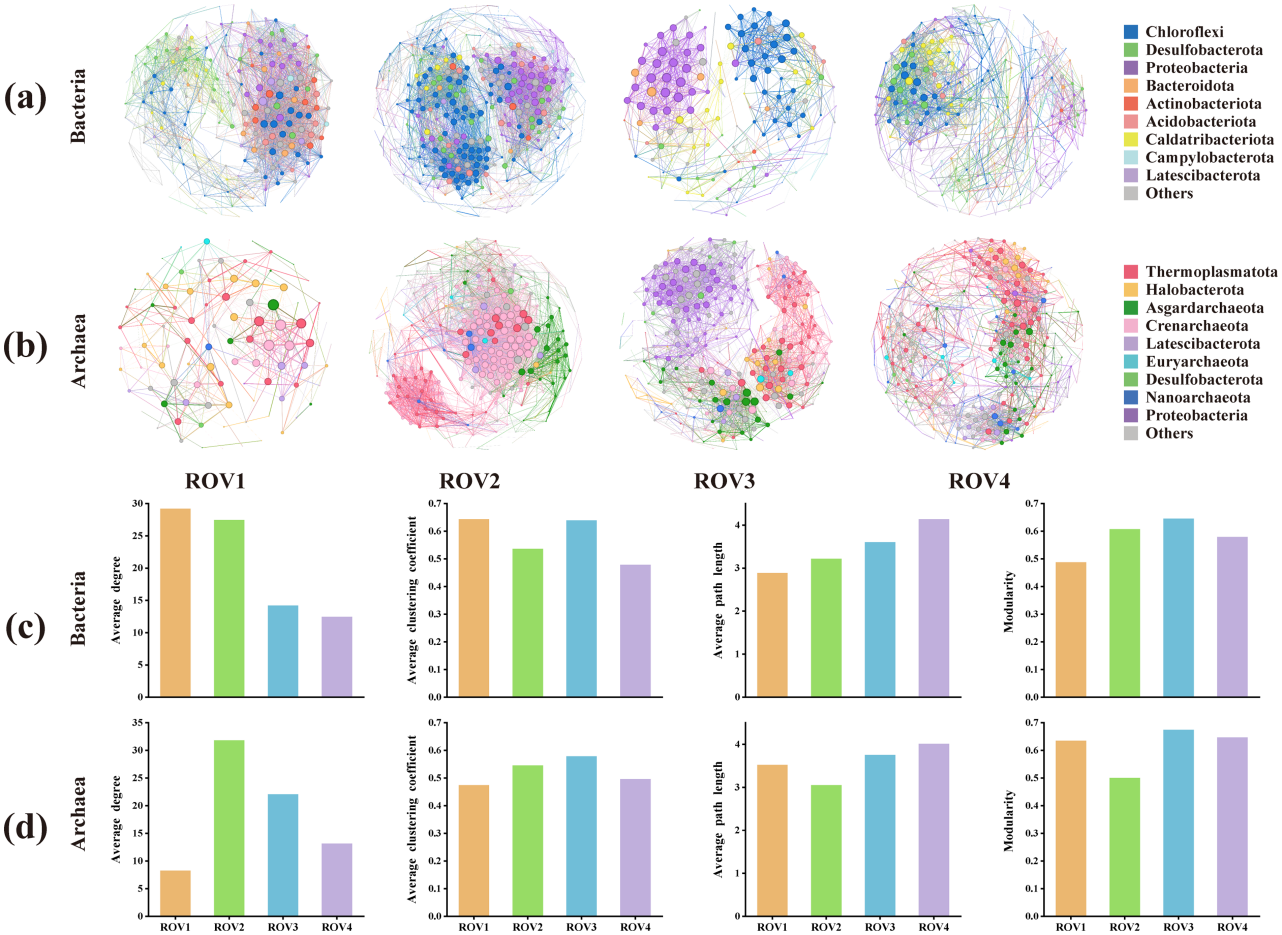
co-occurrence patterns of amplicon sequence variants (ASVs) at sites ROV1, ROV2, ROV3, and ROV4 in networks and links of topological parameters with sites; co-occurrence networks of **(a)** bacterial and **(b)** archaea communities at different sites. Different colors represent different phyla, lines represent connections between microbes, each node represents an ASV, and node sizes represent the proportion of ASVs of the phylum; network topological parameters (average degree, average clustering coefficient, average path length and modularity) of co-occurrence of **(c)** bacterial and **(d)** archaea communities at different sites.

The co-occurrence networks and topological properties are constructed to assess microbial associations (Figure 6). The bacterial co-occurrence network at the ROV1 site exhibited more connections than those at other sites (Figure 6a). In the archaeal community (Figure 6b), the microbial network of ROV1 had fewer nodes and connections when compared with other sites.

The majority of nodes in the bacterial networks were classified within Chloroflexi, Desulfobacterota, and Proteobacteria, whereas the archaeal networks were primarily composed of Thermoplasmatota, Halobacterota, Asgardarchaeota, and Crenarchaeota. The topological properties of the prokaryotic networks were different across sites (Figures 6c,d). The bacterial network at ROV1 exhibited a higher average degree (more connections per node) compared to other sites, suggesting more frequent potential interactions among bacterial species. In contrast, the archaeal network at ROV1 showed the lowest average degree. Furthermore, the network at ROV3 displayed higher clustering coefficient and modularity, indicating tighter cluster formation and greater structural stability of the microbial community at this site.

## Discussion

Differences in biogeological parameters play crucial roles in the formation of distinct biological communities (Zhang et al., 2015; Li et al., 2021; Zhai et al., 2022). Both environmental conditions and physical distance are important factors affecting microbial community structure (Martiny et al., 2006). The four sites in this study exhibited different methane concentrations, with ROV1 exhibiting particularly strong methane seepage. Methane and hydrogen sulfide of seepage sustain key functional groups in the cold seep system, including methanotrophs, methanogens, hydrocarbon degraders, and SRB (Zhai et al., 2022). The flux and duration of seepage are also the crucial drivers of faunal colonization (Seabrook et al., 2018). ROV1 was in a stage of strong methane seepage, where the dominant microbial processes are AOM and sulfate reduction (Figure 1). In this study, ANME-1b, ANME-2c, and SRB such as Anaerolineaceae and SEEP-SRB1 were the dominant microorganisms in ROV1, which exhibited a relatively low *α*-diversity. Meanwhile, the other three sites were in the stages of weaker methane seepage (Figure 1), where diverse overlying fauna thrived by relying on chemotrophic microorganisms (Feng et al., 2018). Consequently, the relatively higher α-diversities were observed at the other three sites. The sample aggregation and site distance displayed by PCoA further confirmed the discrepancies between microbial communities of different cold seep habitats.

As the results showed, Desulfobacterota was a highly abundant phylum and the SEEP-SRB1 of this phylum was predominant in bacterial communities at ROV1. Halobacterota was a predominant phylum in the archaeal communities, particularly at ROV1, where ANME-1b and ANME-2c were the dominant groups. The symbionts with ANME-2c or ANME-1 are widely distributed in cold seep habitats (Kleindienst et al., 2012). The relatively high methane content and representative microorganisms of ROV1 suggested that ANME-1b and ANME-2c might collaborate with SEEP-SRB1 to perform AOM. Chloroflexi was also an abundant phylum in bacterial communities and Dehalococcoidia was the dominant class within this phylum at ROV2. Studies have shown that some strains of Dehalococcoidia are associated with the organic degradation in marine sediments or organic dehalogenation (Wasmund et al., 2014; Löffler et al., 2015). Asgardarchaeota was an abundant phylum in the archaeal community of ROV2, and class Lokiarchaeia within this phylum was the main representative microorganism. Lokiarchaeia (Spang et al., 2015) was first discovered in sediments near the Loki’s Castle active vent site. Many members of the Asgardarchaeota phylum including Lokiarchaeia were regarded as anaerobic fermentative heterotrophs involved in the sediment carbon cycle (Busi et al., 2021). A study has found that a representative strain of Lokiarchaeia (*Candidatus* Prometheoarchaeum syntrophicum MK-D1) from cold seep sediments grew syntrophically with a methanogen and SRB and degraded amino acids and peptides (Imachi et al., 2020). Thermoplasmatota was also an abundant phylum in archaeal communities at ROV2, ROV3, and ROV4. Studies have shown that some members of this phylum are host-associated or free-living methanogens (order Methanomassiliicoccales) or may be involved in the degradation of organic matter in marine sediments (*Candidatus* Yaplasmales) (Borrel et al., 2020; Zheng et al., 2022).

In summary, Desulfobacterota and Halobacterota predominated at ROV1 where relatively strong sulfate reduction and anaerobic oxidation of methane occurred. Chloroflexi and Asgardarchaeota were relatively abundant in sediments of ROV2, suggesting that there might be microbial degradation of organic matter. Accordingly, the environmental similarity of ROV3 and ROV4 corresponds to the fact that both sites were dominated by Proteobacteria and Halobacterota. In general, divergent environmental conditions led to the heterogeneous composition of the prokaryotic community.

The discrepancies in geochemical parameters, such as CH_4_, TIC, SO_4_^2−^, and Ca^2+^, might be the key drivers of the differences in microbial communities among various cold seep habitats. In hydrate-bearing ecosystems, it can be reasonably hypothesized that CH_4_ can affect microorganisms. Several studies have shown that methane seepage is correlated with the structure of the surrounding microbial community (Zhang et al., 2012; Li et al., 2021). Seepage fluids from cold seeps shaped microbial communities in these environments, especially anaerobic methanotroph communities (Vigneron et al., 2019). The aggregation of microorganisms in cold seep sediment had obvious biogeographical distribution characteristics, which were related to in-situ geochemical conditions, such as the concentrations of methane and sulfate (Heijs et al., 2007; Jiang et al., 2022). Methanotrophic microorganisms are often coupled with SRB, which are thought to be the main force involved in the production and consumption of sulfate (Xu et al., 2020). HCO_3_^−^ combines with Ca^2+^ to form carbonate rocks, which consume calcium ions. The significantly lower concentrations of SO_4_^2−^ and Ca^2+^ at the ROV1 site suggested an intensification of AOM at this site (Chen et al., 2023). Furthermore, the variations in environmental factors and microbial communities across sites indicated that parameters such as CH_4_, TIC, SO_4_^2−^, and Ca^2+^ concentrations were correlated with microbial community structure. Seepage activity in the Haima cold seeps region varied spatiotemporally (Xu et al., 2020). Consequently, the intensity of seepage differed among sites, thus fundamentally shaping the prokaryotic taxa and functional community structures through variations in energy and nutrient supply (Chen et al., 2023).

Environmental parameters differed among sites and these differences affected the assembly processes of the prokaryotic communities. The interaction of methane-related substances was an important factor that promoted microbial aggregation. The assembly of microbial communities is generally affected by stochastic and deterministic factors, and they synergistically affect the community diversity (Soininen and Graco-Roza, 2024). In this study, stochastic and deterministic processes showed different response balances in prokaryotic communities at different sites. The bacterial community at ROV1 was more affected by stochastic processes (homogenizing dispersal and undominated) than those at the three other sites. Stochastic processes became more vital to community assembly in highly productive environments (Chase, 2010). The high dispersal rate in the region leads to the homogenization of the community and the formation of similar community composition in different areas (Zhou and Ning, 2017). The assembly of the archaeal community at ROV1 was primarily driven by homogeneous selection. In highly productive environments, the colonization of dominant species tends to result in the establishment of populations with a very stable equilibrium (Chase, 2010). Anaerobic methanotrophs such as ANME-1b and ANME-2c were the dominant group at ROV1, and the community composition was relatively simple, with low biodiversity. This might be due to the high content of nutrients such as methane in ROV1, which was conducive to the growth of specific microorganisms. Homogeneous environmental conditions are likely to favor similar species composition and community structure at various sites (Wu et al., 2021). In the samples of sites ROV2, ROV3, and ROV4, the contributions of stochastic and deterministic processes to community assembly were comparable. Furthermore, the contribution of homogeneous selection to community assembly increased with methane content. Microorganisms in these communities adapted to the competitive pressure caused by the environment (Dai et al., 2017).

In short, the heterogeneity of the horizontal nutrient distribution in four sites drove distinct prokaryotic community assembly. At the methane seep site, bacterial community assembly was governed primarily by stochastic processes, whereas archaeal assembly was predominantly deterministic. In contrast, the prokaryotic communities at the faunal sites were influenced by a combination of both processes.

The heterogeneity of environmental factors across sites drove distinct species coexistence patterns in prokaryotic communities. The environment created ecological niches that allowed for the interaction and reproduction of dominant species, playing a crucial role in structuring microbial communities (Freilich et al., 2018). The higher average degree and shorter average path length at ROV1 indicated tight bacterial interactions. In contrast, the archaeal community at ROV1 exhibited significantly lower average degree. This might result from environmental selection pressure exerted by the cold seep fluid. This selective pressure, in conjunction with specific microbial interactions, played a critical role in shaping the microbial community structure and the resultant ecological network at this site (Kraft et al., 2015). Highly abundant taxa are more frequently located in the center of the network (Zhang et al., 2020). The high connectivity and central network position of the major bacteria species at ROV2 suggested their uniformity and irreplaceable roles in the community. The short average path length and medium modularity in the archaeal community at ROV2 suggested widespread microbial co-occurrence and interaction.

To sum up, the ROV1 site exhibited a unique microbial co-occurrence pattern compared to other sites. The bacterial community at ROV1 had a greater number of coexisting species and demonstrated higher connectivity and potential interactions. In contrast, under a strong methane supply, archaeal communities exhibited significantly low species coexistence. Strong methane seepage affected species diversity and their interaction, resulting in different species coexistence patterns of the archaea and bacterial communities. The differences in the other site environments were not significant, but slight differences were still observed in the co-occurrence process. Microbial coexistence exhibited distinct patterns between strong methane seepage areas and faunal habitats, primarily shaped by gradients in methane content. Environmental differences initially drove the aggregation of prokaryotes, selected for suitable species in the ecosystem, and facilitated the further development of different coexisting networks through evolutionary processes such as competition. The influence of the environment on the development of the biological community reflected the adaptation and evolutionary succession of prokaryotes to different habitats in the extreme environment of cold seeps.

## Conclusion

Through a comprehensive investigation of sediment microorganisms from different sampling sites in the Haima cold seeps, differences were found in prokaryotic community assembly and species coexistence patterns among the four sampling sites. Significant differences in the abundance and community structure of microorganisms were found in regions with different methane content. Analysis of *β*-NTI showed that the assembly process of prokaryotic communities at each site was mainly controlled by stochastic processes. Specifically, the archaeal communities in areas with high methane content were driven by the same energy source, and the assembly process also exhibited a significant degree of determinacy. Horizontal environmental heterogeneity affected the diversity and structure of prokaryotic communities, driving the assembly process of bacteria and archaea. Different co-occurrence networks further showed the diversity of species coexistence patterns in different environments. In conclusion, the present study explored environmental heterogeneity observed at different locations in the cold seep from a microbiological perspective, deepening our understanding of how environmental heterogeneity played an important role in the assembly of prokaryotic communities and species coexistence.

## Data Availability

The data presented in this study are publicly available. This data can be found at: https://www.ncbi.nlm.nih.gov/sra, accession number PRJNA1230284.
