# Supplementary material for: Heterogeneity of the horizontal environment drives community assemblages and species coexistence of prokaryotic communities in cold seep sediments
Source: Front Microbiol. 2025 Nov 20;16:1687453. doi: 10.3389/fmicb.2025.1687453 (PMC12675211; doi:10.3389/fmicb.2025.1687453)
Supplement: Supplementary file 1 [file Supplementary_file_1.docx]

**Supplementary material for**

**Heterogeneity of the horizontal environment drives community assemblages and species coexistence of prokaryotic communities in cold seep sediments**

Qixuan Wu^a^, Jingchun Feng^a,b,^^[[1]](#footnote-2)^*, Yongji Huang^a,b,c,d^, Song Zhong^a^, Cun Li^a,b,c,d^, Si Zhang^a,b,d^

^a^ Guangdong Basic Research Center of Excellence for Ecological Security and Green Development, Guangdong University of Technology, Guangzhou 510006, China

^b^ Southern Marine Science and Engineering Guangdong Laboratory (Guangzhou), Guangzhou, 511458, P. R. China

^c^ University of Chinese Academy of Sciences, Beijing 100049, China

^d^ South China Sea Institute of Oceanology, Chinese Academy of Sciences, Guangzhou 510301, P. R. China

Table S1 Geochemical characteristics of surface sediments at sites ROV1, ROV2, ROV3, and ROV4 in Haima cold seeps of the South China Sea. Units used: Depth (cmbsf), CH_4_ (μmol/g), TIC (μg/mg), TOC (μg/mg), SO_4_^2–^ (mmol/L), Cl^–^ (mmol/L), Ba (mmol/L), K (mmol/L), Ca (mmol/L), Mg (mmol/L), Fe (mmol/L), Cu (mmol/L), Mn (mmol/L).

| Sites | Depth | CH_4_ | TIC | TOC | SO_4_^2–^ | Cl^–^ | Ba | K | Ca | Mg | Fe | Cu | Mn |
| --- | --- | --- | --- | --- | --- | --- | --- | --- | --- | --- | --- | --- | --- |
| ROV1 | 5 | 9.46 | 194.01 | 26.40 | 12.80 | 587.03 | 1.0297 | 9.7013 | 3.5349 | 0.0346 | 0.0000 | 0.0000 | 0.0000 |
|  | 10 | 9.72 | 288.82 | 35.77 | 6.22 | 594.16 | 0.9113 | 7.8233 | 4.3790 | 0.0369 | 0.0032 | 0.0000 | 0.0000 |
|  | 15 | 16.13 | 269.15 | 34.04 | 2.80 | 593.10 | 0.4588 | 8.6931 | 2.8476 | 0.0370 | 0.0029 | 0.0000 | 0.0000 |
|  | 20 | 12.95 | 288.98 | 29.98 | 2.58 | 592.43 | 0.3199 | 8.9269 | 2.9501 | 0.0404 | 0.0000 | 0.0000 | 0.0000 |
|  | 25 | 12.43 | 258.49 | 31.27 | 3.04 | 579.89 | 0.2919 | 7.4708 | 2.2721 | 0.0343 | 0.0002 | 0.0000 | 0.0000 |
|  | 30 | 16.49 | 287.01 | 33.63 | 2.29 | 580.00 | 0.1734 | 7.5307 | 2.2989 | 0.0350 | 0.0000 | 0.0000 | 0.0000 |
|  | 35 | 15.44 | 214.16 | 30.13 | 1.83 | 565.58 | 0.1678 | 8.0396 | 1.4748 | 0.0354 | 0.0000 | 0.0000 | 0.0000 |
| ROV2 | 5 | 2.04 | 38.21 | 30.21 | 29.28 | 564.00 | 0.0840 | 8.3327 | 8.0891 | 0.0404 | 0.0021 | 0.0000 | 0.0017 |
|  | 10 | 1.70 | 38.32 | 31.78 | 29.39 | 572.95 | 0.1227 | 9.9059 | 9.4845 | 0.0453 | 0.0001 | 0.0000 | 0.0066 |
|  | 15 | 1.62 | 41.14 | 32.15 | 28.09 | 570.17 | 0.1156 | 10.1312 | 5.2335 | 0.0438 | 0.0011 | 0.0000 | 0.0000 |
|  | 20 | 1.88 | 44.62 | 35.06 | 25.55 | 503.82 | 0.0438 | 5.5182 | 4.4938 | 0.0247 | 0.0023 | 0.0000 | 0.0006 |
|  | 25 | 1.87 | 46.09 | 33.05 | 25.67 | 526.88 | 0.0447 | 7.0982 | 5.7440 | 0.0301 | 0.0000 | 0.0000 | 0.0000 |
|  | 30 | 1.99 | 50.45 | 33.08 | 25.71 | 551.17 | 0.0000 | 9.9711 | 8.9391 | 0.0445 | 0.0000 | 0.0000 | 0.0000 |
|  | 35 | 1.68 | 43.14 | 32.56 | 25.76 | 567.55 | 0.0000 | 6.9964 | 10.7178 | 0.0398 | 0.0000 | 0.0000 | 0.0000 |
| ROV3 | 5 | 3.67 | 40.65 | 30.64 | 29.43 | 586.74 | 0.0926 | 7.3345 | 6.6594 | 0.0325 | 0.0030 | 0.0001 | 0.0037 |
|  | 10 | 2.99 | 42.06 | 30.85 | 29.77 | 587.32 | 0.0621 | 8.1535 | 6.9214 | 0.0355 | 0.0000 | 0.0000 | 0.0031 |
|  | 15 | 3.88 | 44.45 | 33.05 | 29.22 | 589.96 | 0.0638 | 8.3565 | 7.2413 | 0.0374 | 0.0001 | 0.0000 | 0.0000 |
|  | 20 | 4.78 | 55.7 | 34.17 | 27.51 | 579.25 | 0.0946 | 9.1199 | 7.7792 | 0.0409 | 0.0009 | 0.0000 | 0.0000 |
|  | 25 | 5.23 | 68.73 | 34.13 | 28.16 | 602.56 | 0.0996 | 8.5446 | 7.4883 | 0.0382 | 0.0006 | 0.0000 | 0.0000 |
|  | 30 | 6.16 | 67.79 | 35.43 | 27.51 | 587.61 | 0.0907 | 8.8276 | 7.1826 | 0.0398 | 0.0006 | 0.0001 | 0.0000 |
|  | 35 | 7.19 | 50.14 | 44.53 | 26.67 | 575.43 | 0.0833 | 9.4478 | 6.6999 | 0.0400 | 0.0000 | 0.0000 | 0.0000 |

Table. S1 Geochemical characteristics of surface sediments at sites ROV1, ROV2, ROV3, and ROV4 in Haima cold seeps of the South China Sea (continued).

| Sites | depth | CH_4_ | TIC | TOC | SO_4_^2–^ | Cl^–^ | Ba | K | Ca | Mg | Fe | Cu | Mn |
| --- | --- | --- | --- | --- | --- | --- | --- | --- | --- | --- | --- | --- | --- |
| ROV4 | 5 | 5.60 | 46.76 | 51.5 | 28.96 | 556.08 | 1.3830 | 11.5432 | 7.8413 | 0.0477 | 0.0388 | 0.0003 | 0.0000 |
|  | 10 | 4.96 | 45.20 | 38.51 | 27.22 | 521.37 | 0.1168 | 7.4343 | 6.4890 | 0.0344 | 0.0000 | 0.0000 | 0.0000 |
|  | 15 | 6.00 | 46.84 | 31.60 | 28.91 | 558.80 | 0.1888 | 9.9734 | 8.8418 | 0.0437 | 0.0002 | 0.0000 | 0.0000 |
|  | 20 | 6.59 | 57.23 | 34.90 | 27.49 | 535.23 | 0.2316 | 8.5742 | 7.3870 | 0.0390 | 0.0000 | 0.0000 | 0.0000 |
|  | 25 | 6.64 | 71.54 | 36.22 | 29.26 | 578.13 | 0.3167 | 9.7110 | 8.7006 | 0.0427 | 0.0004 | 0.0000 | 0.0000 |
|  | 30 | 6.72 | 84.24 | 38.95 | 28.35 | 556.28 | 0.2378 | 8.1749 | 7.1035 | 0.0372 | 0.0007 | 0.0000 | 0.0000 |
|  | 35 | 5.87 | 84.54 | 39.45 | 29.46 | 582.45 | 0.4165 | 10.2077 | 8.5250 | 0.0444 | 0.0000 | 0.0000 | 0.0000 |

Table. S2 α-diversity of prokaryotic community in surface sediments at sites ROV1, ROV2, ROV3, and ROV4 in Haima cold seeps in the South China Sea.

|  | Sites | Depth | Good coverage | Chao1 | Observed species | PD whole tree | Shannon |
| --- | --- | --- | --- | --- | --- | --- | --- |
| Bacteria | ROV1 | 5 | 0.9963 | 3323.50 | 3192 | 242.41 | 8.82 |
|  |  | 10 | 0.9963 | 3055.98 | 2919 | 226.45 | 8.57 |
|  |  | 15 | 0.9969 | 2383.32 | 2254 | 193.46 | 7.81 |
|  |  | 20 | 0.9979 | 1779.44 | 1709 | 160.56 | 7.23 |
|  |  | 25 | 0.9976 | 1811.84 | 1730 | 172.41 | 7.05 |
|  |  | 30 | 0.998 | 1781.81 | 1716 | 160.55 | 7.19 |
|  |  | 35 | 0.9974 | 1727.00 | 1615 | 161.49 | 6.29 |
|  | ROV2 | 5 | 0.9928 | 6011.12 | 5757 | 351.27 | 10.76 |
|  |  | 10 | 0.9896 | 6938.10 | 6504 | 355.50 | 10.87 |
|  |  | 15 | 0.9951 | 4924.10 | 4752 | 296.95 | 10.28 |
|  |  | 20 | 0.9925 | 5463.42 | 5154 | 306.91 | 10.37 |
|  |  | 25 | 0.9947 | 4878.43 | 4686 | 302.19 | 10.19 |
|  |  | 30 | 0.9941 | 5311.15 | 5110 | 318.55 | 10.41 |
|  |  | 35 | 0.9932 | 5100.56 | 4811 | 309.44 | 10.17 |
|  | ROV3 | 5 | 0.9923 | 6347.63 | 6072 | 350.37 | 10.68 |
|  |  | 10 | 0.9913 | 5635.00 | 5260 | 333.66 | 9.97 |
|  |  | 15 | 0.9994 | 2281.23 | 2272 | 196.73 | 8.89 |
|  |  | 20 | 0.9991 | 3424.69 | 3410 | 270.75 | 9.80 |
|  |  | 25 | 0.9988 | 3587.12 | 3567 | 275.26 | 9.67 |
|  |  | 30 | 0.9993 | 2430.11 | 2417 | 206.20 | 8.78 |
|  |  | 35 | 0.9988 | 3307.15 | 3287 | 263.28 | 9.16 |
|  | ROV4 | 5 | 0.9992 | 3671.00 | 3660 | 256.33 | 10.33 |
|  |  | 10 | 0.9991 | 2720.80 | 2708 | 229.71 | 9.31 |
|  |  | 15 | 0.9981 | 3930.92 | 3885 | 294.15 | 10.13 |
|  |  | 20 | 0.9983 | 3634.20 | 3595 | 261.63 | 9.93 |
|  |  | 25 | 0.9991 | 3325.91 | 3312 | 246.65 | 10.10 |
|  |  | 30 | 0.9986 | 3491.56 | 3463 | 263.01 | 9.76 |
|  |  | 35 | 0.9975 | 3999.17 | 3929 | 280.33 | 10.35 |

Table. S2 α-diversity of prokaryotic community in surface sediments at sites ROV1, ROV2, ROV3, and ROV4 in Haima cold seeps in the South China Sea (continued).

|  | Sites | Depth | Good coverage | Chao1 | Observed species | PD whole tree | Shannon |
| --- | --- | --- | --- | --- | --- | --- | --- |
| Archaea | ROV1 | 5 | 0.9988 | 547.98 | 517 | 74.54 | 2.14 |
|  |  | 10 | 0.9985 | 559.01 | 510 | 75.50 | 2.25 |
|  |  | 15 | 0.9983 | 729.99 | 684 | 75.15 | 3.81 |
|  |  | 20 | 0.9993 | 363.04 | 352 | 43.10 | 2.71 |
|  |  | 25 | 0.9991 | 348.84 | 324 | 38.29 | 2.52 |
|  |  | 30 | 0.9993 | 293.07 | 270 | 34.46 | 2.33 |
|  |  | 35 | 0.9990 | 432.28 | 411 | 48.70 | 2.89 |
|  | ROV2 | 5 | 0.9989 | 2044.14 | 2034 | 149.29 | 8.53 |
|  |  | 10 | 0.9984 | 1760.32 | 1738 | 141.30 | 8.05 |
|  |  | 15 | 0.9973 | 2083.68 | 2034 | 166.75 | 8.05 |
|  |  | 20 | 0.9986 | 1312.05 | 1288 | 104.97 | 7.28 |
|  |  | 25 | 0.9979 | 1421.50 | 1379 | 125.71 | 7.14 |
|  |  | 30 | 0.9991 | 713.28 | 696 | 87.39 | 5.26 |
|  |  | 35 | 0.9994 | 740.45 | 732 | 71.59 | 5.33 |
|  | ROV3 | 5 | 0.9999 | 1705.19 | 1705 | 159.24 | 7.41 |
|  |  | 10 | 0.9997 | 939.28 | 938 | 105.13 | 6.25 |
|  |  | 15 | 0.997 | 1478.88 | 1389 | 108.81 | 6.28 |
|  |  | 20 | 0.9965 | 1784.78 | 1678 | 136.82 | 6.70 |
|  |  | 25 | 0.9968 | 1996.37 | 1909 | 133.66 | 7.59 |
|  |  | 30 | 0.9978 | 1170.17 | 1106 | 92.18 | 5.06 |
|  |  | 35 | 0.9962 | 1718.00 | 1597 | 117.67 | 6.40 |
|  | ROV4 | 5 | 0.9971 | 2293.26 | 2235 | 198.19 | 8.28 |
|  |  | 10 | 0.9978 | 1632.70 | 1578 | 138.50 | 6.64 |
|  |  | 15 | 0.9972 | 1584.41 | 1517 | 127.34 | 6.19 |
|  |  | 20 | 0.9968 | 1325.37 | 1231 | 109.02 | 5.18 |
|  |  | 25 | 0.9966 | 1821.23 | 1738 | 153.05 | 7.11 |
|  |  | 30 | 0.996 | 1942.19 | 1834 | 145.19 | 6.95 |
|  |  | 35 | 0.9954 | 1987.01 | 1841 | 148.51 | 7.03 |

Table S3 Topological features of co-occurrence network of prokaryotic communities in surface sediments at sites ROV1, ROV2, ROV3, and ROV4 of Haima cold seeps in the South China Sea.

|  | Sites | Average degree | Average clustering coefficient | Average path length | Modularity |
| --- | --- | --- | --- | --- | --- |
| Bacteria | ROV1 | 29.144 | 0.642 | 2.878 | 0.486 |
|  | ROV2 | 27.403 | 0.535 | 3.207 | 0.606 |
|  | ROV3 | 14.144 | 0.638 | 3.593 | 0.644 |
|  | ROV4 | 12.401 | 0.477 | 4.127 | 0.578 |
| Archaea | ROV1 | 8.198 | 0.473 | 3.513 | 0.634 |
|  | ROV2 | 31.739 | 0.544 | 3.04 | 0.499 |
|  | ROV3 | 21.993 | 0.577 | 3.745 | 0.673 |
|  | ROV4 | 13.071 | 0.495 | 4.002 | 0.646 |


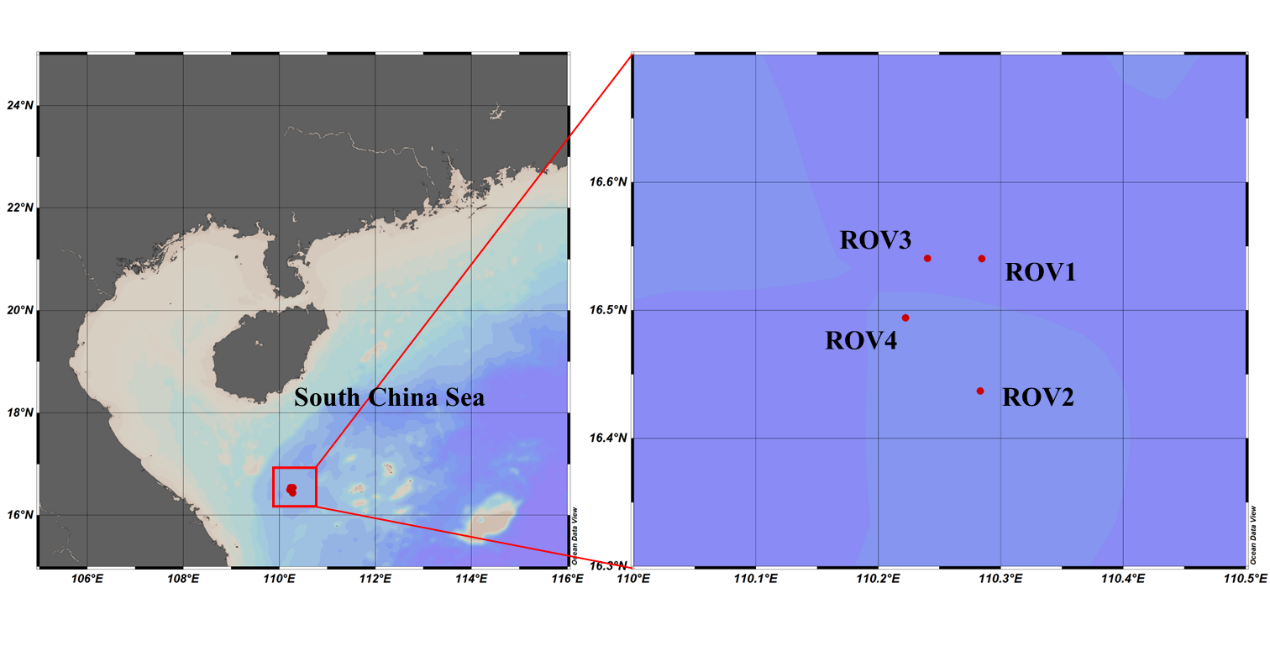


Fig. S1 The sampling sites distribution of the Haima cold seep in the South China Sea in this study. The four sampling sites are ROV1 with methane seepage, and ROV2, ROV3, and ROV4 mainly colonized by mussels, clams, and sea anemones, respectively.


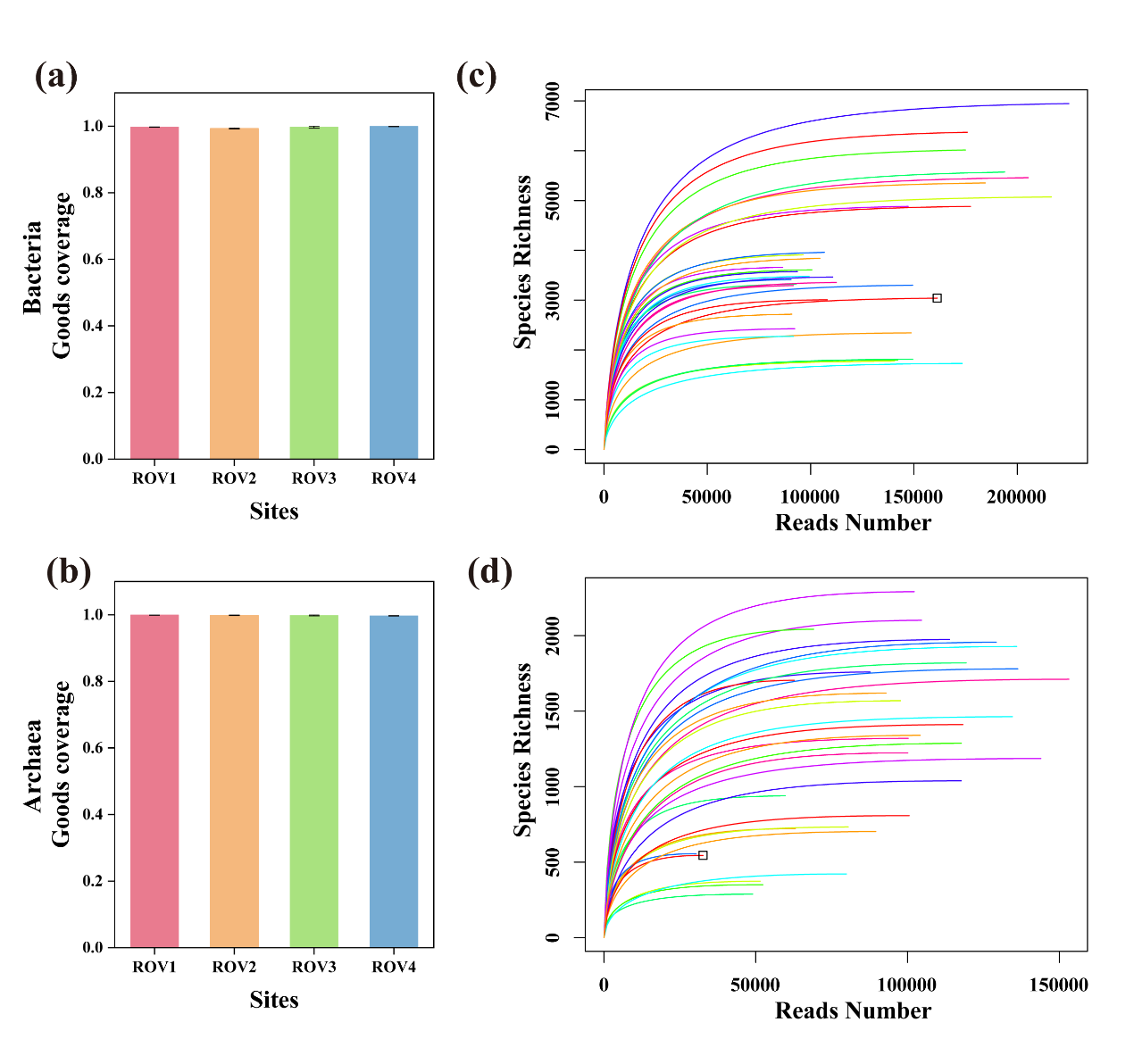
Fig. S2 Good coverage index of (a) bacteria and (b) archaea at sites of Haima cold seeps in the South China Sea. Rarefaction curves of (c) bacteria and (d) archaea.


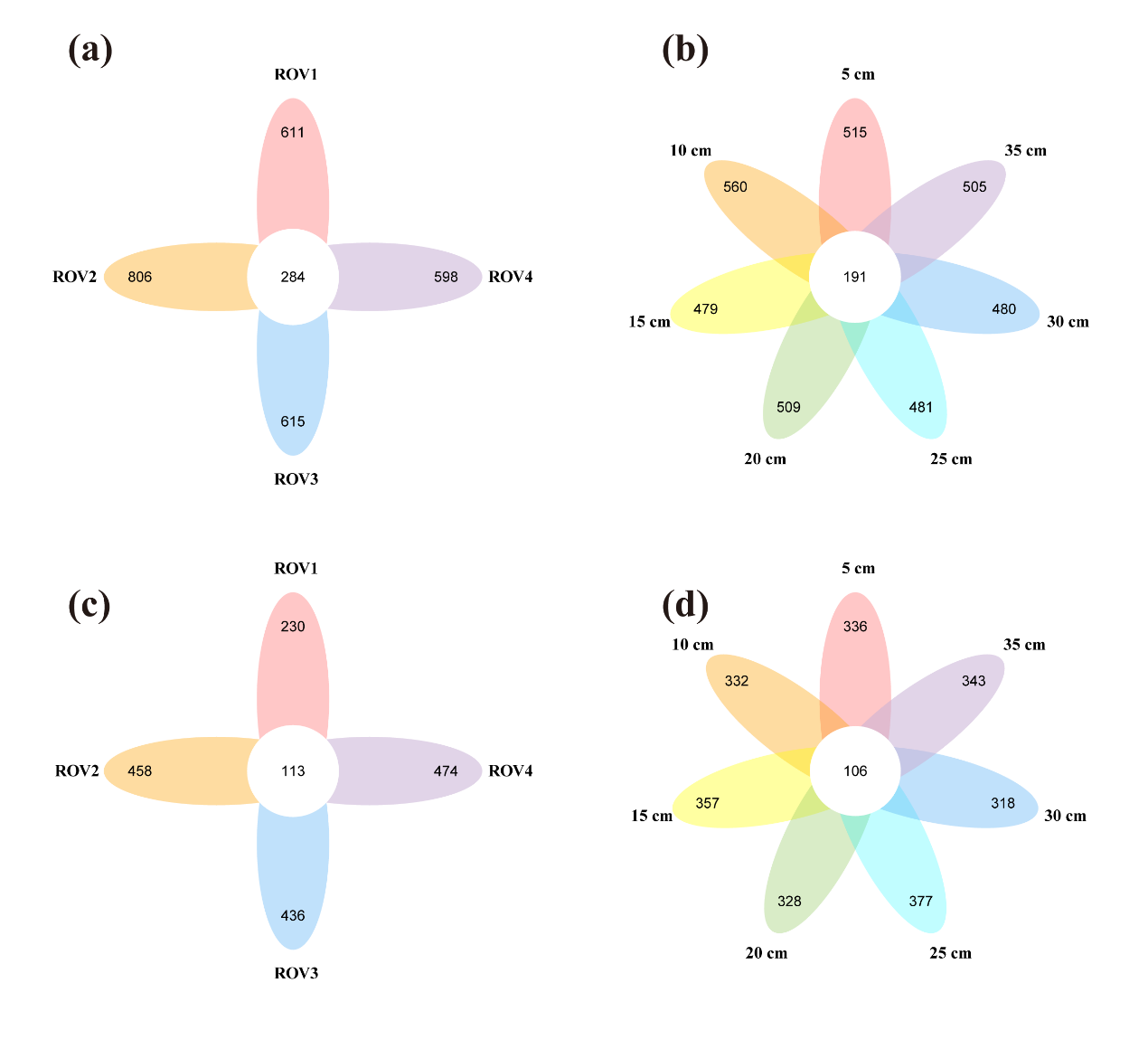
Fig. S3 Flower plots showing the number of shared and non-shared operational taxonomic units of bacteria grouped by (a) site and (b) depth, and archaea grouped by (c) site and (d) depth. Note: ROV1, ROV2, ROV3, and ROV4 are site numbers.


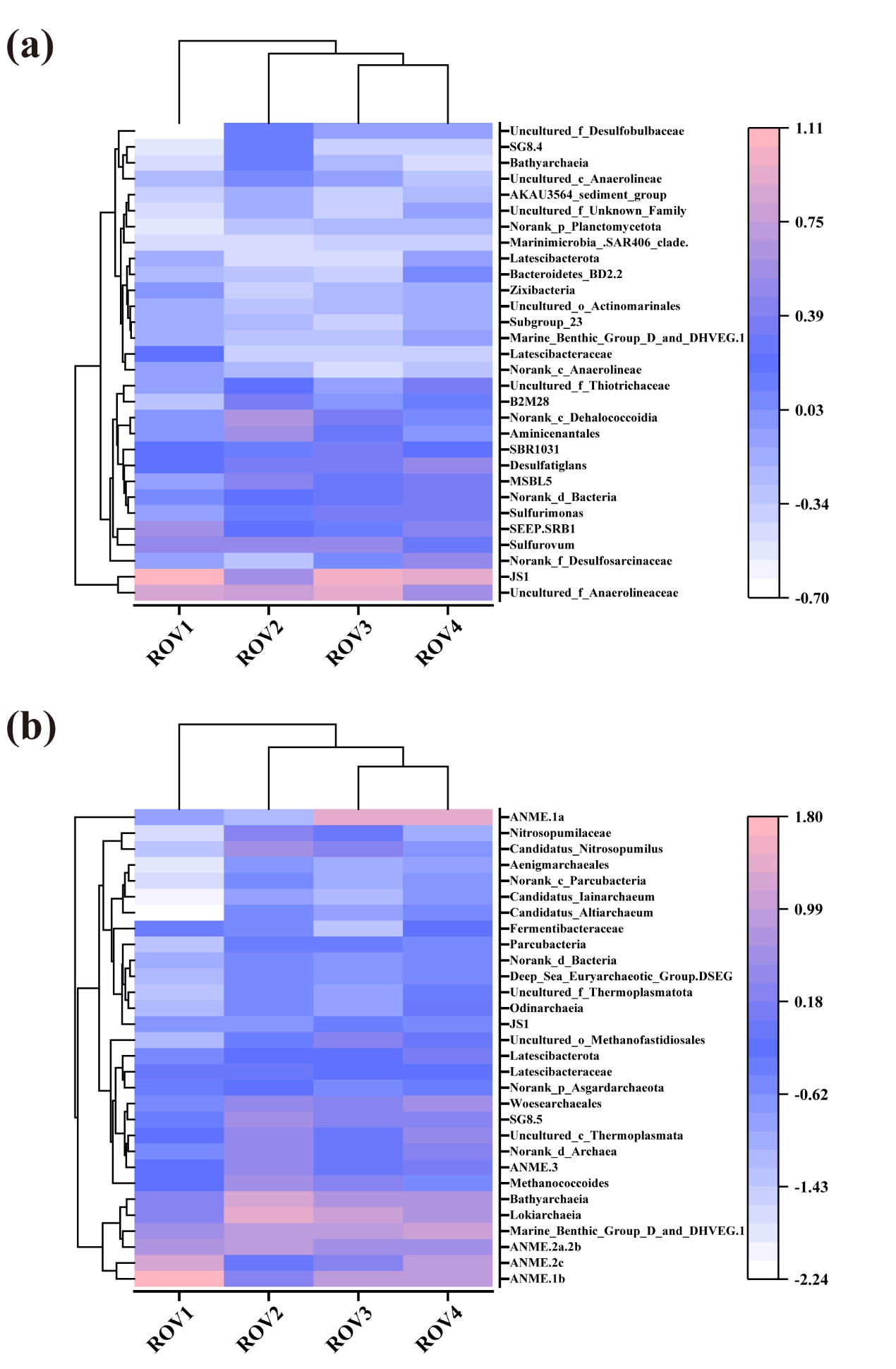
Fig. S4 Heatmaps of differences in (a) bacterial and (b) archaeal community composition based on 30 most abundant taxa at sites ROV1, ROV2, ROV3, and ROV4 of Haima cold seeps in the South China Sea.

1. [↑](#footnote-ref-2)
